# Recent Advances in Polymer Flooding in China

**DOI:** 10.3390/molecules27206978

**Published:** 2022-10-17

**Authors:** Kaoping Song, Jianwen Tao, Xiuqin Lyu, Yang Xu, Shaopeng Liu, Zhengbo Wang, Huifeng Liu, Yuxuan Zhang, Hongtao Fu, En Meng, Mingxi Liu, Hu Guo

**Affiliations:** 1 School of Petroleum Engineering, Northeast Petroleum University, Daqing 163318, China; 2 Unconventional Petroleum Research Institute, China University of Petroleum-Beijing, Beijing 102249, China; 3 No.1 Oil Production Plant, Sinopec Northwest Oil Field Company, Urumqi 830011, China; 4 Geological Research Institute, China National Logging Company Ltd., Xi’an 710077, China; 5 CNOOC EnerTech-Drilling & Production Company, Tianjin 300450, China; 6 Oilfield Chemistry Department, CNPC Research Institute of Petroleum Exploration and Development, Beijing 100083, China; 7 Overseas Engineering Technology Research Institute, CNPC Engineering Technology R&D Company Limited, Beijing 102206, China; 8 Wells Engineering Department, CNPC R&D (DIFC) Company Limited, Dubai 415747, United Arab Emirates

**Keywords:** polymer flooding, EOR, residual oil saturation, viscosity, polymers, field tests

## Abstract

Polymer flooding is drawing lots of attention because of the technical maturity in some reservoirs. The first commercial polymer flooding in China was performed in the Daqing oilfield and is one of the largest applications in the world. Some laboratory tests from Daqing researchers in China showed that the viscoelasticity of high molecular weight polymers plays a significant role in increasing displacement efficiency. Hence, encouraged by the conventional field applications and new findings on the viscoelasticity effect of polymers on residual oil saturation (ROS), some high-concentration high-molecular-weight (HCHMW) polymer-flooding field tests have been conducted. Although some field tests were well-documented, subsequent progress was seldom reported. It was recently reported that HCHMW has a limited application in Daqing, which does not agree with observations from laboratory core flooding and early field tests. However, the cause of this discrepancy is unclear. Thus, a systematic summary of polymer-flooding mechanisms and field tests in China is necessary. This paper explained why HCHMW is not widely used when considering new understandings of polymer-flooding mechanisms. Different opinions on the viscoelasticity effect of polymers on ROS reduction were critically reviewed. Other mechanisms of polymer flooding, such as wettability change and gravity stability effect, were discussed with regard to widely reported laboratory tests, which were explained in terms of the viscoelasticity effects of polymers on ROS. Recent findings from Chinese field tests were also summarized. Salt-resistance polymers (SRPs) with good economic performance using produced water to prepare polymer solutions were very economically and environmentally promising. Notable progress in SRP flooding and new amphiphilic polymer field tests in China were summarized, and lessons learned were given. Formation blockage, represented by high injection pressure and produced productivity ability, was reported in several oil fields due to misunderstanding of polymers’ injectivity. Although the influence of viscoelastic polymers on reservoir conditions is unknown, the injection of very viscous polymers to displace medium-to-high viscosity oils is not recommended. This is especially important for old wells that could cause damage. This paper clarified misleading notions on polymer-flooding implementations based on theory and practices in China.

## 1. Introduction

**Figure 1:**
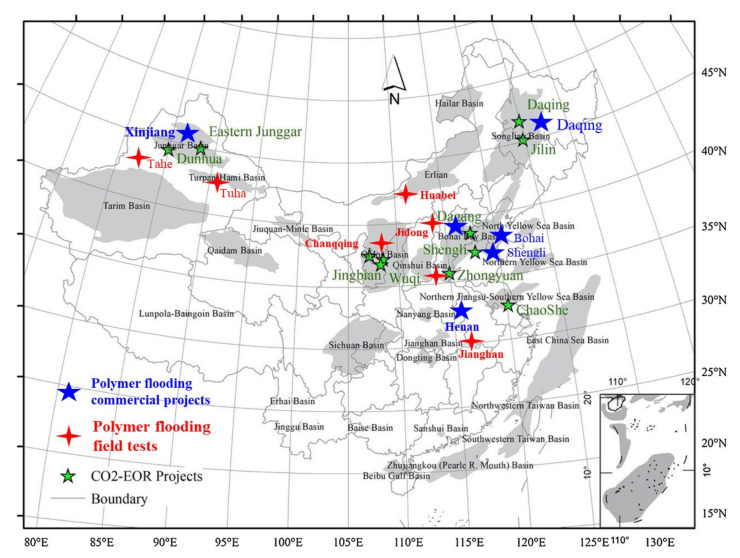
Polymer flooding in China (revised after map from [11]).

Polymer flooding is a mature chemical enhanced oil recovery (EOR) technology that has been widely commercialized in both onshore and offshore reservoirs [1]. Although polymer flooding was first field-tested in the USA, the largest field tests and commercial use were first reported in China [2]. Figure 1 shows the worldwide distribution of polymer-flooding projects, of which 119 projects are in China. However, according to a recent survey in 2018 [3], there are more than 170 polymer-flooding projects in China and 93 active commercial field blocks in the Daqing oilfield. This indicated that some polymer-flooding advances were unknown to international readers. Recent studies indicated that the incremental oil recovery factor (IORF) of polymer flooding upon water flooding in some blocks in Daqing was 15.51% of the original oil in place (OOIP), with a total oil recovery of 59.67% of the OOIP [4]. This high IORF value is significant considering that the average oil recovery of all oilfields is approximately one third of the OOIP [5]. Although many polymer-flooding projects were conducted in sandstone reservoirs, both with light and heavy oils, carbonate reservoirs were also field-tested [6]. The progress on polymer flooding in Daqing has been well-documented. However, polymer flooding in other Chinese oilfields, including in Shengli [7,8], Henan [9], and Xinjiang [10], is also worth noting because of their harsher reservoir conditions. In addition to the Daqing oilfield, polymer flooding has also been commercially used in Shengli, Henan, Dagang, Xinjiang, and Bohai oilfields, as well as in field tests in other oilfields, as shown in Figure 1. Figure 1 was revised from the CO_2_ EOR projects in China’s sedimentary depositional basins from [11]. The figure shows that although polymers have been widely used in many Chinese oilfields, their use is still under contention because a large number of reservoirs, including very viscous oil (Liaohe) and high-temperature high-salinity (HTHS) (Zhongyuan, Tarim) and low-permeability reservoirs (Changqing, Jilin), in China do not commercially use polymer flooding. Figure 1 also shows that polymer-flooding projects have been able to compete with CO_2_ EOR projects in the past two decades. Although CO_2_ EOR projects were field-tested in many Chinese oilfields, their commercial application remained limited and of small scale. To ensure carbon neutrality, CO_2_ EOR could be developed at an accelerated pace. However, a large number of problems still exist in China. Insufficient and affordable CO_2_ was one cause to account for the limited CO_2_ EOR oil production. Only a small number of the CO_2_ EOR projects indicated in Figure 1 were commercially applied. Approximately 3% of the annual oil production from CO_2_ projects came from polymer flooding as of 2020 [12]. Recent advances in CO_2_ EOR were reviewed in [12]. Despite the progress that was made in applying polymer flooding to very viscous oils in the Pelican Lake in Canada [13,14,15,16,17] and the Alaska North Slope in the USA [17,18,19,20,21,22,23,24], the selection of polymer viscosity and slug size to recover very viscous oils [25,26,27,28,29] remains quite different from that in China because of the not well-understood mechanisms under an unfavorable mobility ratio. Polymers have been regarded as not having an effect on residual oil saturation (ROS) reduction [5,30]. However, many researchers in China theorized that polymers could reduce ROS. To this end, high-concentration high-molecular-weight (HCHMW) polymer-flooding field tests have been conducted [31,32,33]. Recently, the viscoelasticity effect of polymers on ROS has been extensively researched [34,35,36,37,38,39]. However, ROS reduction reported from core flooding tests could also be caused by mechanisms other than polymer viscoelasticity. The findings and observations of field practices require further investigation. In addition to the benefits of EOR, polymer flooding was reported to significantly reduce the carbon emission intensity [40,41] partly because of invalid and saved water injections, which avoided to produce CO_2_ emission. Hence, polymer flooding will be more popular in the face of carbon emission reduction and carbon neutrality. Thus, the theory and practical aspects of polymer flooding in China have been summarized. Notably, a large number of publications (exceeding 7000) and some good review papers [1,42,43,44,45] regarding polymer flooding have been published in the past 30 years. Therefore, this review presented a theoretical and practical summary from a different perspective to reduce cost and avoid the risk of possible polymer-flooding applications.

## 2. Theoretical Aspects

Chinese research on polymer flooding is focused on the viscoelasticity effect of polymers on ROS reduction and, consequently, on the displacement efficiency, polymer matching criteria with porous media, and synthesis of new polymers with improved viscosity capacity compared with typical hydrolyzed polyacrylamides (HPAMs). The blockage of injectors in some oilfields, such as the offshore SZ 36-1 [46] in the Bohai oilfield, is becoming increasingly concerning. This problem is especially serious for high-concentration polymer-injection blocks in oilfields such as Daqing, Xinjiang, and Henan. The blockage evaluation method is regarded as a matching-up theory between polymers and porous media parameters, including permeability and pore size. The actual reservoir pressure distribution could be of concern. As modern chemical EOR is based on the maximum capillary number and (practically) minimum mobility ratio theory, the notion of combining the benefits of reducing interfacial tension (IFT) and increasing the displacing phase viscosity leads to the synthesis of an amphiphilic polymer, which has the features of both polymers and surfactants. This new type of polymer is commonly referred to as a polymeric surfactant in China. Chemical EOR is closely related with the capillary number. Previous studies have exhibited the problems of classic capillary numbers and potential benefits of revised capillary numbers [47,48]. A critical review of capillary numbers and their applications in EOR was presented in previous studies [49,50].

### 2.1. Viscoelasticity Effect of Polymers on Residual Oil Saturation Reduction

**Conventional wisdom**. It has long been believed that the viscoelasticity of polymers has no or an insignificant effect on ROS reduction. This is reflected in the Society of Petroleum Engineers (SPE) textbook, Enhanced Oil Recovery, which was first published in 1998 and revised in 2018 ([5], p.5). In another SPE textbook, it was assumed that the elastic properties of polymers did not result in a lower ROS ([30], p.306). The reasons against the effect of polymers on ROS reduction are summarized as follows:

(1) ROS can be quantitatively correlated with the capillary number, which is the ratio of the viscous to capillary force. Polymers cannot increase the capillary number to a critical value, the desired content of which is at least three orders of magnitude [49,51]. Polymers have a negligible effect on the oil–water IFT, and, therefore, no reduction of trapped oil, which behaves similarly to ROS, is expected compared with water flooding [26].
(2) Polymers do not have a significant effect on oil phase relative to permeability [25]. Thus, it cannot reduce ROS. Laboratory core flooding tests are well-simulated based on the assumption that the relative permeability curve would be the same as that of water flooding [52]. However, there are different opinions regarding the relative permeability curve [53].
(3) Homogeneous core flooding tests were used to show that polymers do not have an effect on the reduction of ROS [54]. The reported core flooding tests indicate that ROS reduction using polymers is due to heterogeneity that caused improved sweep efficiency rather than displacement efficiency [39]. Compared with water flooding, the main function of polymers is to enlarge the sweep efficiency in the low and medium layers. In addition, it was determined that the viscoelasticity effect of polymers on ROS is limited [55].
(4) It has also been concluded from some laboratory core flooding tests that ROS reduction can be caused by artifacts [54]. It is difficult to accurately read the oil volume at high water cuts, such as 99%. Extreme long-term water (injected water at 10,000 PV) flooding indicates that the displacement efficiency can be increased from 45% to 80% when the termination water cut is decreased from 98% to 99.5% [56].
(5) The micromodel experimental results demonstrated that even if the viscoelasticity of the polymer is large, its effect on ROS is negligible in terms of saturation changes [57]. The recovery difference between a high and low viscoelastic polymer flood in the micromodels was 2%, which was regarded as insignificant [58].
(6) The recent research [56] regarding polymer flooding in the Daqing oilfield, where the largest commercial polymer flooding was conducted and the largest HCHMW was tested, showed that the HCHMW was not widely employed despite being successfully implemented in many field tests wherein good performance was reported. This has been discussed in detail in [59].

**Figure 2:**
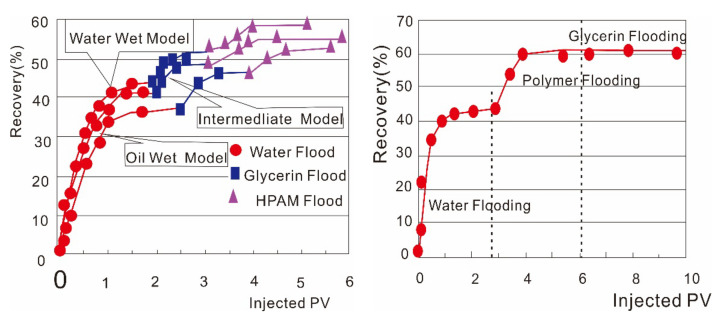
Typical core flooding tests in favor of viscoelasticity effect (revised from [61]).

**Major findings**. When Daqing researchers in China first reported that polymers could reduce ROS because of its viscoelasticity [60,61,62,63], many field tests were conducted [31,32,33]. In China, [60] first reported that polymers could reduce ROS through laboratory and field tests. When more larger amount of polymer is injected, polymer-flooding performance in the Daqing oilfield was much better than that of earlier pilots at lower polymer amounts, as seen in [1]. In [1], a yearly increase in the polymer-injection volume can be noted. It can be noted that the oil recovery factor incrementally increased with an increase in the polymer-injection volume. The scattered data can be attributed to factors such as the remaining oil saturation before polymer injection, oil–water viscosity ratio, and operation level. Thus, further research can be performed on increased polymer-injection volumes with higher polymer concentrations and molecular weights (MWs). HCHMW polymer-flooding tests with a large scale were reported to have an incremental oil recovery factor (IORF) of 10% of the original oil in place (OOIP) recovery compared with conventional polymers [31,32,33]. Polymer core flooding tests under typical Daqing reservoir conditions are shown in Figure 2 [32]. From these results, it was theorized that polymers could recover oil that cannot be displaced using glycerin. However, this mechanism may not be significant if the density difference between the oil droplets and water is considered. Moreover, glycerin and HPAM are not entirely comparable because glycerin is a small molecule and HPAM is a long-chain polyelectrolyte [64]. More importantly, the use of glycerin in combination with brine could yield erroneous results due to strong dependence of the glycerin viscosity on mixing with water [65].

**Figure 3:**
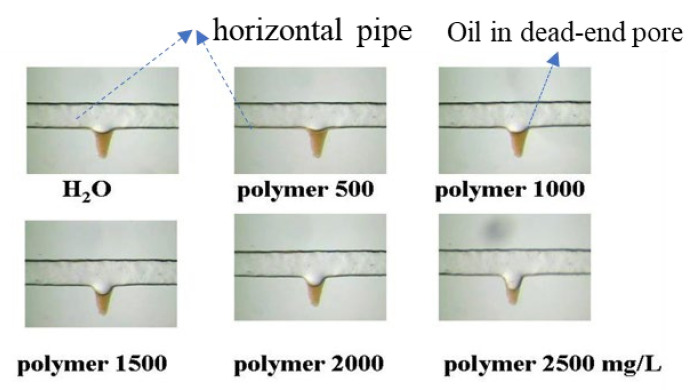
Dead-end oil displaced by polymer (revised from [32]).

Figure 3 [32] shows dead-end oil that was remobilized using polymers and is used to account for the viscoelasticity effect mechanism. In Figure 3, “polymer 500” means the concentration of the polymer solution was 500 mg/L. For abbreviation, the unit was given only once in Figure 3. It could be concluded that the higher polymer concentration displaced more dead-end residual oil. However, the displaced residual oil is more likely to be displaced by mechanisms other than the believed viscoelasticity effect. Sun et al. [66] performed experiments using both water and oil wet pores. For water wet pore models, which were similar to those in [32], the viscoelasticity effect did not appear to affect the dead-end residual oil [66]. In this reference, a significant increase in the viscosity of polymers was observed, which typically corresponds to an increase in viscoelasticity according to previous studies.

**Figure 4:**
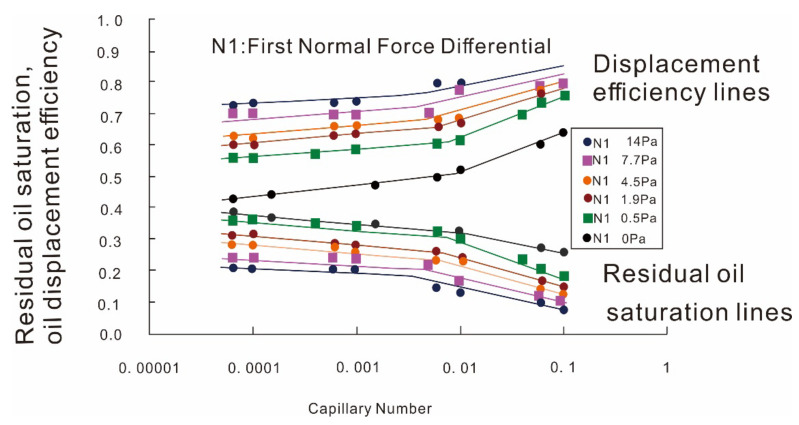
Viscoelasticity affected capillary desaturation curve (revised after [32]).

The capillary desaturation curves (CDCs) shown in Figure 4 [32] were used to account for the viscoelasticity effect of polymers on ROS reduction. In Figure 4, the lower group indicates ROS, whereas the upper group indicates the oil displacement efficiency corresponding to ROS. “N1” refers to the first normal stress difference of the polymers, which reflects their viscoelasticity. Other parameters were kept constant. CDCs are well-suited to describing the correlation between ROS and the capillary number. However, there are some problems with traditional capillary number theory [47,49,51,67,68]. The proposed explanations are brought into question by the fact that the viscoelasticity and normal stress differences disappear at low velocities [26]. Hence, other mechanisms may exist.

**Figure 5:**
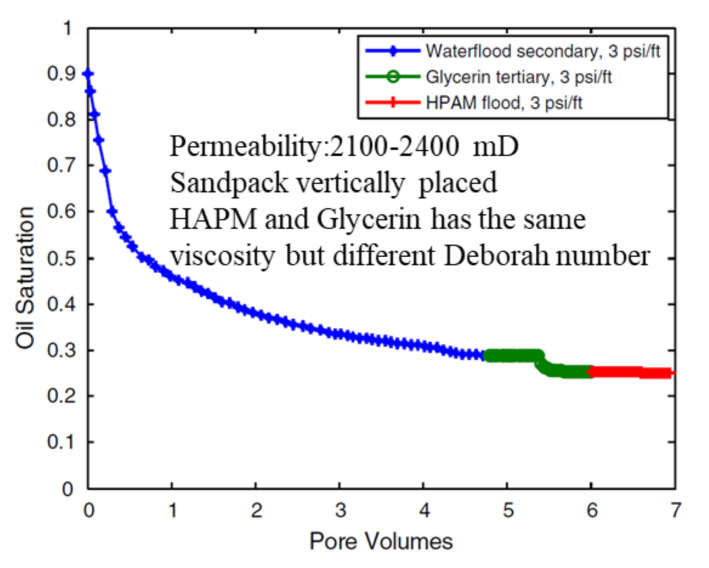
Polymer effect on residual oil saturation (revised after [39]).

Recently, many researchers have attributed reported ROS reduction to the viscoelasticity of polymers [35,39,69,70]. A typical test result is shown in Figure 5 [39], which is significantly different from a classic CDC. This is because the capillary number was not significantly increased, but ROS was significantly reduced. In other words, without attaining the critical capillary number, which typically required the addition of surfactants to significantly reduce the IFT, ROS can be significantly reduced. A critical review [34] on the viscoelastic effects during polymer flooding highlighted the importance of extensional viscosity in porous media. However, these explanations require further investigation because of their inconsistency with laboratory studies [25] and field practices [13,14,15] of polymer flooding in very viscous reservoirs in North America.

### 2.2. Other Potential Mechanisms

**Wettability change**. Wettability changes could account for some of the observations regarding ROS reduction in the laboratory. HPAM can reduce the contact angle from 97.52° to 75.93° [71]. Yue et al. [72] reported that HPAM changed the wettability of quarts to be more water-wet after being soaked. The water static and HPAM imbibition rates are different, but the ultimate oil recovery is similar to that of water-wet cores [73]. However, dynamic imbibition with fractures is more complex. The addition of polyacrylamide increased the number of hydrophilic and hydrophobic functional groups adsorbed onto the kaolinite surface, with a larger increase noted for hydrophobic groups compared with hydrophilic groups [74]. The presence of adsorbed polymers increases the water irreducible saturation of the core [75]. HPAM-based colloidal dispersion gel (CDG) injection changed the rock wettability to be more water-wet [76]. The moderate oil wettability was changed to weak water wettability by using a polymer solution based on the contact angle tests [77]. Zeta potential tests performed on different types of brine and natural sand showed that the addition of polymers to low-salinity water (LSW) had a negligible effect on the electrical charge on the sand surface. The Zeta potential changed from −17.78 mV for the LSW to −18.4 mV for the low-salinity polymer flooding (LSP) [20], indicating a slight wettability change toward water-wet due to the addition of polymers. However, it should be addressed that these tests [20] were carried out in the absence of oil. Thus, the effect of viscoelasticity on dead-end oil droplets shown in Figure 3 [32] can also be caused by wettability changes. Notably, brine without viscoelasticity can also displace dead-end oil [64,78]. Although core flooding tests showed that the branched polymers with slightly higher elasticity had a higher IORF [64], a clear effect of elasticity on oil recovery for the linear polymers could not be determined from the two-dimensional flow-cell tests [79]. It has also been reported that higher viscoelasticity polymers have lower oil recovery from dead-end zones [79].

**Gravity stability effect**. The gravity difference between water and oil shown in Figure 3 [32] could result in the production of dead-end oil if the flooding time is long enough. If the model in Figure 3 [32] was turned over, the results could have differed because of the gravity effect. The use of the gravity effect to counter viscous fingers was demonstrated in a gravity-stable surfactant flooding test, wherein the core was vertically placed and without the addition of polymers to control the mobility [80]. Although the experiments were partially attributed to the in situ emulsions formed by the surfactants in a previous study [80], horizontal surfactant flooding was unstable and did not demonstrate the same benefits as the in situ emulsions. The gravity stability effect was more obvious in high-permeability formation than in low-permeability formation because the effect of gravity outweighed the capillary and viscous forces. The relative importance of gravity, capillary forces, and viscous forces was studied by [81].

**Low-salinity effect**. It is also possible that the low salinity used in polymer flooding is an intrinsic recovery mechanism, as proposed by [30]. It is notable that the brine salinities in laboratory polymer-flooding core tests and field tests in Daqing were much lower than in seawater or other high-salinity formation waters in other oilfields. Hence, the synergy between the LSW and polymer flooding could also account for some of the test results. However, this low-salinity effect was not extensively researched other than with regard to its influence on polymer solution effects. The synergy effect of LSW with polymer flooding was demonstrated through core flooding tests [82]. LSP could improve oil recovery by 8% after high-salinity polymer flooding [82]. It was also proven that LSP could synergistically increase oil recovery compared with low-salinity and conventional polymer flooding.

**Other key observations**. A critical review [34] of the viscoelastic effects during polymer flooding highlighted the importance of the extensional viscosity in porous media. However, further investigation is required because of an inconsistency with the laboratory studies [25] and field practices [13,15] on polymer flooding in very viscous reservoirs in North America. In another study, hardly any effect was observed on the oil recovery of crude oils with a high viscosity of 300 cP after the viscoelasticity was increased [65]. In other words, if the polymer viscoelasticity could reduce ROS, it is unexpected that the viscoelasticity effect would disappear in a heavy-oil polymer-flooding test. Thus, for high-viscosity crude oils, the viscoelastic effect did not improve oil recovery over polymer floods with low viscoelasticity [36]. Although one may argue that the mobility ratio between polymers and very viscous oils was too large for the effects caused by viscoelasticity to be observed, it is rare that hardly any effect is observed. Further investigation is required to determine whether the viscoelasticity of a polymer can cause a significant effect. Furthermore, no field tests outside of China reported on the contribution of the viscoelasticity of polymers on ROS.

**Field update**. The notion that the viscoelasticity effect influences ROS reduction is widely accepted in China. An improved numerical simulation method with consideration of the viscoelasticity effect of polymers has been proposed and widely used in polymer flooding in Daqing [83,84]. Although the IORF of HCHMW field tests is reportedly as high as 20% of the OOIP and some commercial use was promoted, including in Daqing, it was shown that the HCHMW was not widely used in Daqing [56]. This will be discussed in detail later.

**Practical considerations**. An issue with polymer viscoelasticity is that HPAM retains its viscoelasticity after a high-rate shearing in near-well regions because of the large pressure gradient. When polymers are injected into a reservoir, they suffer significant mechanical degradation [38,85,86]. Daqing researchers commonly believe that the polymer viscosity is reduced by 50% because of near-wellbore mechanical degradation [43]. An offshore oilfield sampling test in China concluded that the viscosity of polymers underground was only a third of the viscosity in the well head [87]. The formation temperature was 62 °C. Interestingly, the MWs of polymers produced in the reservoir were not reduced by more than 50% compared with those that were injected into the reservoir [44]. Viscosity loss during high-pressure pumping, in mixed systems and in the near-wellbore region, is as high as 70% of the total viscosity loss [45]. However, another produced fluid test in a higher-temperature reservoir in the Shengli oilfield indicated that the viscosity loss of a polymer solution 105 m away from the injectors is 66.7% [88]. The test is shown in Table 1 [88]. It is noted that the Shengli oilfield reservoir temperature is 70 °C or higher, whereas the temperature of the Daqing oilfield reservoir is 45 °C. It was expected that the total viscosity loss would have been higher in the Shengli oilfield than in the Daqing oilfield. A study showed that the HPAM polymer coil size decreased from 243.16 to 182.46 nm under a high shearing rate [89]. Based on the results of a DLS test performed on a produced fluid associative polymer, the MW decreased from 12 to 1 MDa and the polymer size decreased from 710–720 to 270–292 nm [90]. The viscoelasticity also increased the in situ viscosity so that it was higher than that of the bulk viscosity under a high shear rate [91,92,93], which could significantly increase the injection pressure. Although this is not regarded as a problem because fracturing often happened [26,94], it increases the number of requirements that the injection system needs to adhere to. An issue with regard to polymer blocking formation is also present. It should be noted that oxidative degradation of HPAM could potentially have a considerable effect on viscosity loss [95]. However, this is not of great concern under the Daqing reservoir condition [96]. It remains unknown if special sampling techniques have been developed that take into account oxidative degradation or the microbe effect, which could seriously affect the viscosity without proper sampling [97]. Recent studies [98,99] indicated that the mechanical degradation of polymers during formation could be overlooked if oxidative degradation was taken into consideration, in which case the actual viscosity loss caused by mechanical degradation could be reduced to less than 50%.

Hence, there are three incidents regarding the viscoelasticity effect of polymers on ROS that require further investigation.

(a) A viscoelastic polymer does not influence the reduction of ROS. In this case, ROS reduction, if there is any, is caused by mechanisms other than the viscoelasticity effect. If the viscoelastic effect of polymers on ROS was considered in oilfield development plans, the oil recovery would be overestimated.
(b) A viscoelastic polymer that has an influence on the reduction of ROS that is too small to be detected at typical low viscosities. This could especially be true in some heavy-oil polymer-flooding projects.
(c) Viscoelastic polymer can play a significant role on reducing ROS. Some technical problems such as contradiction between practical injectivity and expected maximum viscoelasticity effect remain to be resolved, which would explain why it is not commonly used. The oil recovery would be higher than expected if the viscoelasticity effect of polymers on ROS was not considered.

### 2.3. Blockage Evaluation Method

**Figure 6:**
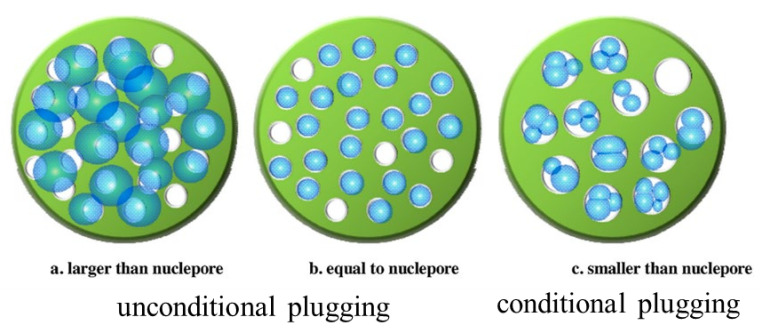
Plugging mechanism of microspheres in nucleopore membranes (revised after [101]).

**Figure 7:**
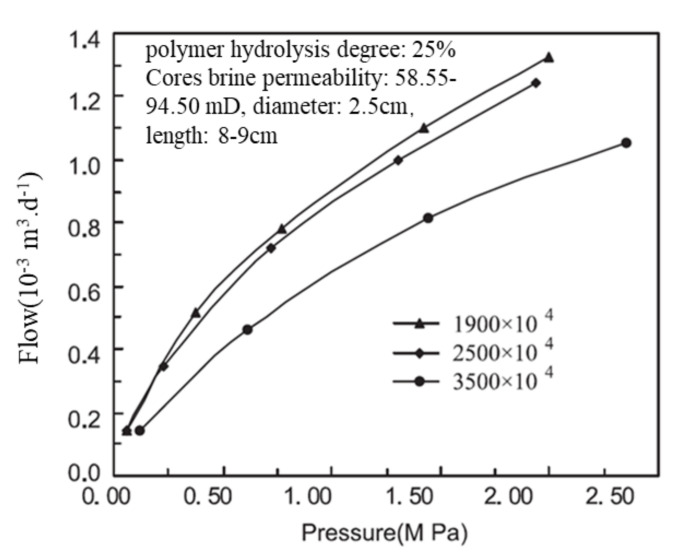
Different polymer molecular-weight flow curves (revised after [104]).

The selection of polymer viscosity and concentration remains an important but challenging issue, especially for HCHMW. The polymer injectivity is often associated with the relationship between the polymer and reservoir rock. According to a survey on polymer-flooding projects, the polymer dosage significantly increased over time, often in terms of concentration times slug (mg L^−1^ PV^−1^) [26]. The IORF by polymer flooding generally increased as the injected polymer dosage increased, as seen in [1]. It should be noted that the IORF results from this reference are closely related to not only the polymer-injection dosage but also the secondary oil recovery. Polymer-flooding field tests in Daqing [43,44,45] indicated that an increase in the polymer-injection volume corresponded to an increase in the IORF. Therefore, the risk of viscous polymers blocking the reservoir when injected should be considered. In the past 20 years, many studies about the relationship between polymers, their pore structures, and reservoir formation have been conducted in China. One such study suggests that polymers be regarded as a hard sphere and to test their size, even though polymers in solutions are not spherical. The key is to fix the polymer coil size. The polymer coil size was characterized using a light-scattering detector [100]. Then, the relationship was determined by comparing the sizes of the polymer spheres and pores. For example, the plugging mechanism between the microsphere and nuclear-pore membrane was introduced, as seen in Figure 6 [101]. In Figure 6, if the microsphere size was smaller than the nuclear-pore size, plugging is still possible, and this is regarded as “conditional plugging”. If the microsphere size was larger than the nuclear-pore size, it is very difficult to pass the membrane, and this is called “unconditional plugging”. Unconditional plugging means the injection should cause very high injection pressure and low injectivity, which should be avoided. Some researchers have attempted to determine the polymer coil size in solutions and determine a relationship between the polymer parameters and permeability [102]. However, this approach is limited by the use of a nucleopore membrane to determine the polymer coil size as it cannot accurately reflect the pore size distribution and the significant effect of the polymer on the pore structure [91,92,93]. In addition, how the homogeneous membrane itself can represent heterogeneous porous media remains to be answered. Their selection of the candidate polymers is potentially limited based on their molecular sizes and the size distribution of the rock pore throats [103]. Therefore, a residual resistance factor (RRF) and resistance factor (RF) could be used to determine whether a particular polymer could block a given core [104]. Alternatively, polymer flow tests could be conducted on the artificial or natural cores [105]. Figure 7 [104] shows the pressure changes of three polymers with different MWs. It was concluded that the pressure gradient is significantly more affected by the polymer concentration than the polymer MW. Researchers that study HCHMW have focused on both the polymer concentration and MW. As previously mentioned, this issue is still under debate as the results are not in agreement. A typical relationship given by the researchers in Daqing is shown in [106]. Many different relationships were identified in [106]. However, the results should be interpreted with care. For example, despite applying similar theories, [107] achieved different outcomes. According to another publication from the Daqing oilfield [108], the injection of polymers with sizes of 8, 12, and 25 MDa corresponded to the formation of polymers with sizes of 35, 100, and 300 mD based on polymer size matching with the average pore size. This indicates that the relationship varied even in Daqing. It is advised that face plugging tests [109] be performed and that cores be used instead of nuclear-pore membranes. It is still under debate whether polymer matching with reservoir formation is affected by the water flood condition [104] because water cut is generally a macroscopic parameter. There exists a detailed review on the current matching theory and Chinese practices [110,111]. It should be reiterated that reservoir conditions, such as pressure and temperature, were not well-simulated in most laboratory tests reported to date.

## 3. Polymer Development and Production

The polymer production technology in China has rapidly developed to meet the increased need. The first pilots in China used polymers imported from foreign countries as they could not be locally produced. Polymer flooding was initially commercially used in Daqing and Shengli in the 1990s, and domestic polymer production technologies have since rapidly developed. Promotion to international corporations encouraged top polymer production companies to build factories in China. Many Chinese companies can now produce high-quality HPAM. At present, China is one of the largest producers of synthetic polymers. The amount of polymers used for EOR in China is much higher than that in any other country. A stable and affordable supply of polymers was crucial to the incorporation of polymer flooding on a commercial scale. In comparison, CO_2_ flooding was not as widely used in China because of the poor availability of an affordable CO_2_ source [12]. Most Chinese oil companies, including those in Daqing, Shengli, and Dagang, can produce their own polymers. In addition to HPAM, a variety of new polymers, including comb-type [112], star-type [113], associative [114,115,116,117,118], biological [119], amphiphilic [120,121,122,123,124,125,126,127], thermoviscosifying [128,129], permeability self-adaptive [130,131], and salt-resistance polymers (SPRs) [132,133,134,135,136], are synthesized and used in field tests and applications. Some polymers have good salinity tolerance under the standard conditions used in Chinese reservoirs. The salinity tolerance criteria for Chinese oilfields may differ from those in foreign companies. The polymers produced outside of China are reviewed in a recent publication [137].

### 3.1. Comb-Shaped Polymer—KYPAM

**Figure 8:**
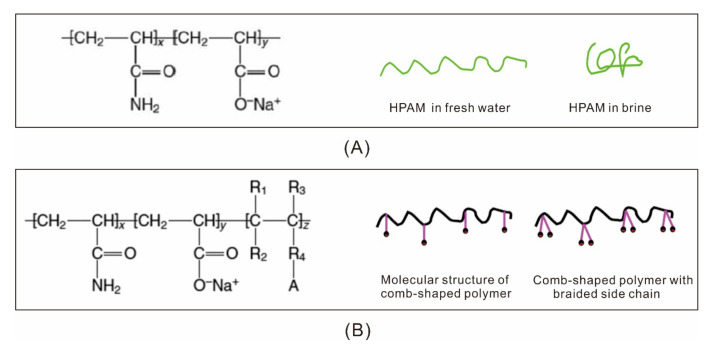
Chemical structure of hydrolyzed polyacrylamide (**A**) and comb-type polymer (**B**) (revised after [112,139]).

The aim of EOR research was to produce polymers with good salt-resistance performance, high viscosity, and good oil displacement capacity during the early stages of polymer flooding. One of the most successful comb-shaped polymers, KYPAM, was first reported in 2002 [138]. The structure of such types of polymer is comb-shaped. Hence, it is referred to as a comb-type polymer. High-quality KYPAM can be produced on an industrial scale. It has good salt-resistance performance and viscosity capacity compared with other major polymers produced in the 1990s, such as HPAM. The structures of ordinary and comb-type polymers are shown in Figure 8 [112]. Figure 8 illustrates the difference in the structure of HPAM in fresh and brine water [139]. Compared with HPAM, KYPAM has both hydrophilic and hydrophobic branches that repel each other, which reduces the possibility of curving and coiling [140]. The MW of KYPAM exceeds 25 MDa, and its viscosity is 30%–40% higher than other ultrahigh MW polymers [138]. Field tests performed in Daqing, Shengli, and Henan using KYPAM verified that less than 30% of KYPAM was required to increase incremental oil production by 4.6 times compared with HPAM [141,142,143].

### 3.2. Amphiphilic Polymer—APP

An amphiphilic polymer refers to a polymer with the capacity to reduce the oil–water IFT and increase the displacing phase viscosity. The amphiphilic polymer APP has been extensively researched in China. This polymer is also known as a polymeric surfactant [127]. However, it has a different structure and performance compared with typical surfactants and polymer–surfactants (SPs) [144]. It is also sometimes inappropriately referred to as an SP [145]. APPs have a reportedly much higher viscosity and larger molecular size than HPAM [146]. The molecular size distributions of these polymers also differ quite significantly because the APP DLS results showed more peaks. About 0.15% APP can reduce the IFT from 30 to 0.1 mN m^−1^ under Daqing oil–water conditions. The IORF of APP flooding in a lab is reportedly 10% and 13% higher than that of HPAM flooding [127]. APPs have increased sweep and displace efficiencies [146]. The molecular size of APPs is large enough that the core surface could easily become blocked. The RF of APPs is much higher than that of HPAM and CDG. In addition, the RFF of APPs is lower than that of CDG and a bit larger than that of HPAM [147]. The molecular structure of APPs is given in [120]. Some researchers have claimed that APPs have a similar structure to HPAM [127,148]. However, other researchers contend that APPs are actually a polymer mixture [120,149,150]. The synthesis and evaluation of APPs are detailed in [120,127,149,150]. Except for their viscosity behavior, APPs have an excellent emulsification capacity, which likely accounts for the increased recovery compared with HPAM [120,127,149,150]. A disadvantage of APPs is their high adsorption into reservoir formation [140]. APP flooding in postpolymer field tests indicated that APPs have a considerably higher retention than conventional polymers [151]. The injectivity of APPs is also of concern because of their large molecular size. The results from a core flooding test [132] showed that the injection pressure of APPs was the highest among SP, ASP, and HCHMW, but that their viscosity was not. Another problem associated with an APP is that it is, at present, still unknown if it consists of one polymer or if it is a weak combination of two different polymers. This combination is different from the traditional HPAM polymer that has different MWs or MW ranges, which could cause instability. Two types of APPs are used and have been field-tested in the Daqing oilfield [145,151,152].

### 3.3. Hydrophobically Associating Water-Soluble Polymer

**Figure 9:**
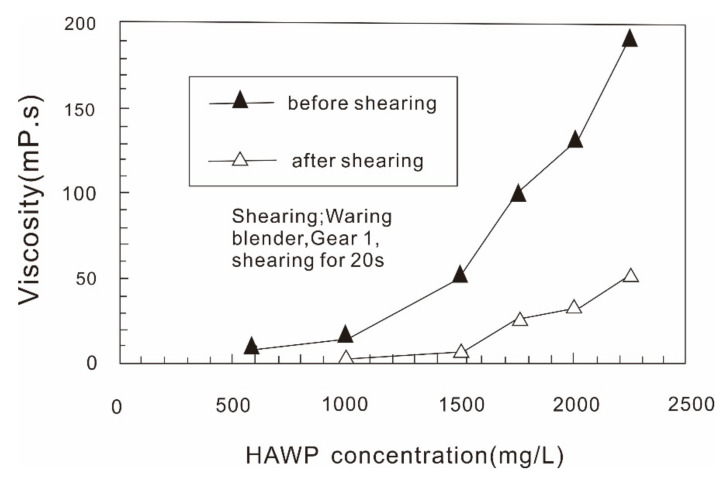
Viscosity–concentration curve under typical reservoir condition [153].

Another commercially used polymer is the hydrophobically associating water-soluble polymer (HAWP) [118,153,154]. This associative polymer is commercially referred to as AP-P4 in China. HAWPs are based on HPAM but have an additional hydrophobic part. They have a longer dissolution time than HPAM because of this hydrophobic part. HAWPs reportedly have better viscosity performance than HPAM [118]. Although the rheological properties of HAWPs have been well-studied and they reportedly have better viscosity than HPAM [114,115,117,155], they are more widely used in offshore reservoirs than in onshore ones. However, HAWPs appear to suffer from a poor antishearing property, as is shown in Figure 9 [153]. Some researchers [156] theorize that the viscosity of HAWPs is able to self-recover after shearing. HAWPs were successfully used in the first offshore polymer-flooding pilot in 2003 and have been commercially used in the largest offshore reservoir in China [118,153]. However, their use is often associated with blockage problems, of which the mechanism is still unclear [157,158]. Although HAWPs were not initially used in the Daqing oilfields when polymer flooding first became commercialized in 1996, their use in ASP flooding has since been considered [56,116]. Some researchers theorize that due to the critical aggregation concentration (CAC), associative polymers do not have good performance when they are diluted using a formation brine [140].

### 3.4. Amphiphilic Polymer—IAJN

**Figure 10:**
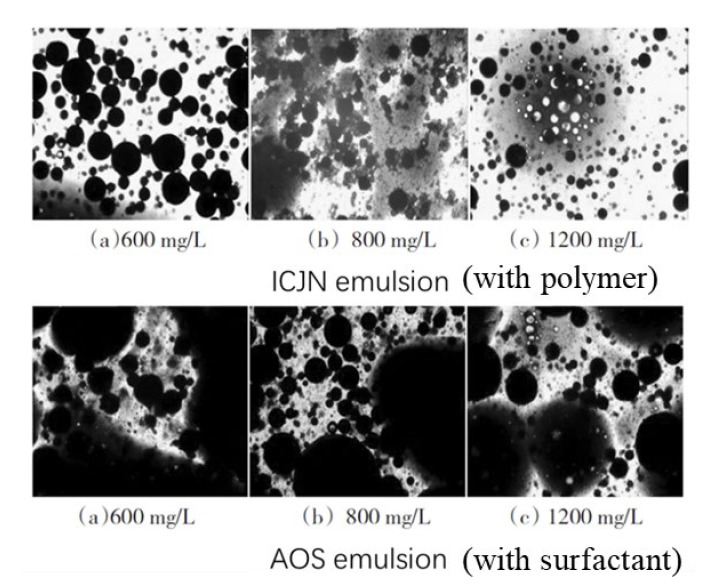
Comparison of emulsification abilities of amphiphilic polymers IAJN and AOS (revised after [124]).

There is another new amphiphilic polymer that was developed for the recovery of offshore heavy oils and has been extensively researched under various names, including ICAJ [121] and ICJN [122,124,159]. This polymer has the ability to activate heavy oils by reducing its viscosity [71]. This polymer is referred to as IAJN hereafter. In contrast to APPs, IAJN can considerably reduce the viscosity of heavy oils [71]. At concentrations of 800–1200 ppm, IAJN can generally reduce the viscosity of heavy oils by 96% [124]. Scanning-electron-microscope pictures showed that IAJN can emulsify the oil to smaller and more uniform emulsions [121]. Although IAJN can reduce the oil–water viscosity to 1 × 10^−1^ mN m^−1^, the CAC of IAJN is only 50 ppm, which is considerably smaller than surfactants with CACs of 1100 ppm [123]. The IAJN aggregation particle size is considerably larger and more stable than that of smaller molecular surfactants [123]. The emulsions formed from IAJN and surfactants are notably different, as is shown in Figure 10 [124]. The results shown in Figure 10 indicate that the emulsification ability of IAJN is considerably better than that of the surfactant. Amphiphilic polymers are adept at peeling off asphaltenes from solid–liquid interfaces [160]. Thus, IAJN could potentially enhance oil recovery using one of the following mechanisms: the emulsification mechanism [71,121,124,161], peeling off of asphaltene from solid–liquid interfaces [160], oil viscosity reduction [71,124,159], wettability alteration [71,123], or interfacial tension reduction [71,123,124]. Although IAJN has a large number of advantages, the high retention because of its structure could affect injectivity and transportation during formation.

It could be concluded that IAJN is not well-suited for EOR. For example, IAJN has a considerably higher adsorption than HPAM, which brings its transportation and formation adsorption abilities into question. Furthermore, if IAJN is used as an EOR polymer, it will need to be modified to meet the screening criteria requirements. Another important feature for an EOR polymer in China is its antishearing capacity, which is often characterized as its viscosity before and after shearing. IAJN has been tested in offshore oilfields.

### 3.5. New Salt-Resistance Polymers

**Figure 11:**
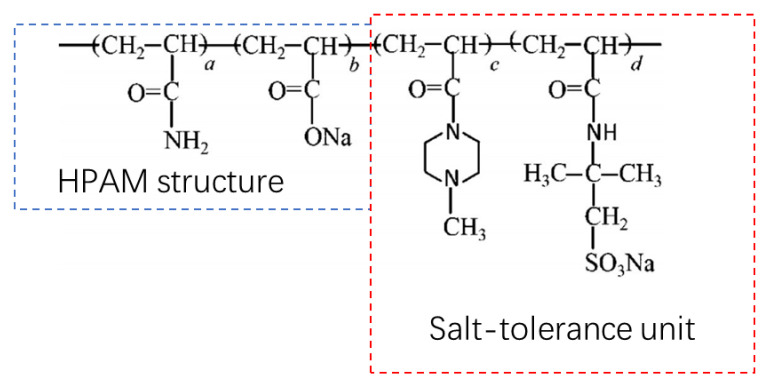
Salt-resistance polymer structure (revised after [133]).

Another notable new polymer that was developed by the Daqing oilfield was the salt-resistance polymer (SRP). KYPAM is a mature SRP that has successfully been used in many Chinese oilfields. However, it was not designed for low-permeability reservoirs, and the need to use produced water to make polymer solutions is daily increasing. Hence, some new polymers were developed to make use of produced water, of which Daqing has a large amount. Three types of SRPs were developed and field-tested in China: DS800, DS2500, and LH2500. The MW of DS800 was 8 MDa, whereas the MW of both DS2500 and LH2500 was 25 MDa. The oil displacement efficiency of DS800 was 4.5% higher than that of previous polymers with the same MW [135]. DS2500 has an improved injectivity compared with conventional HPAM polymers as it has a smaller injection limit of 52 mD compared with the 221 mD of HPAM polymers [135]. This means that the DS2500 polymer can be injected into formations with permeabilities as low as 52 mD, whereas conventional polymers with an MW of 25 MDa can only be injected into formations with permeabilities higher than 221 mD. DS800 and DS2500 were designed for low-permeability reservoirs, and their most prominent feature is that they are self-adaptive [130]. The structure and synthesis of these polymers were elaborated on in [130,131]. Although polymers were injected into some low-permeability cores in laboratory studies, polymer flooding in low-permeability reservoirs has not been frequently reported at field scales. Another SRP called LH2500, which has been field-tested in Daqing, also exhibits better performance than polymers with an MW of 25 MDa [162,163]. However, the origin of these polymers remains unknown. The SRPs have been field-tested in Daqing and exhibit good economic performance. Another newly developed salt-resistant polymer named Polymer “A”, which targets low-permeability formations in the transition zone area in Daqing, is shown in Figure 11 [133]. This polymer was prepared with produced water and was successfully field-tested in Daqing [133]. It remains unknown whether Polymer “A” is DS800 or DS2500. Notably, both DS800 and DS2500 have been produced on an industrial scale according to [164]. DS800 and DS2500 can reduce the polymer amounts used in field applications in Daqing by 20% and 10%, respectively [164].

### 3.6. Thermoviscosifying Polymer

**Figure 12:**
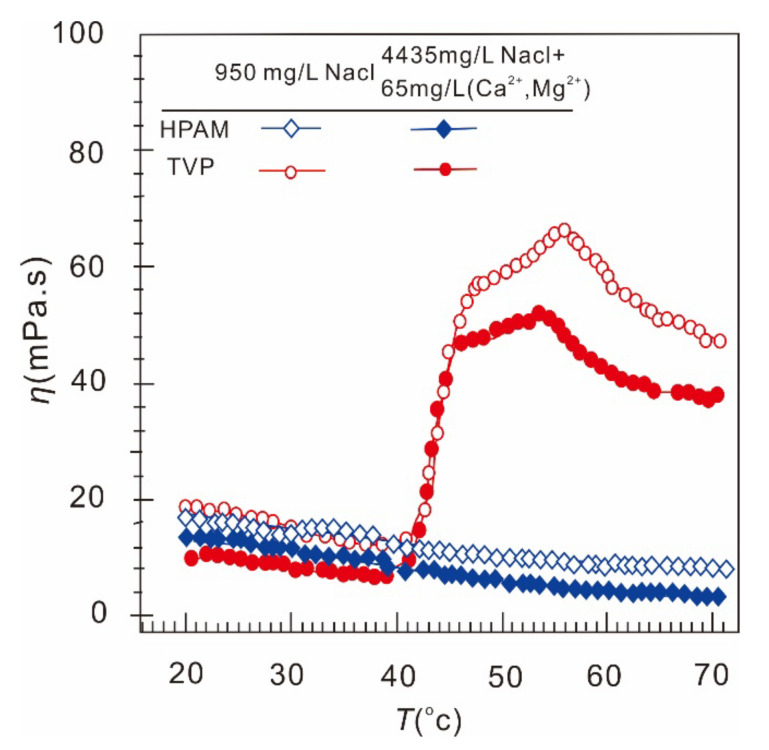
Viscosity of thermoviscosifying polymers in comparison with hydrolyzed polyacrylamide [128].

A prospective thermoviscosifying polymer (TVP) that can be used for low-permeability formation was developed by the Daqing oilfield and its partners. Under typical Daqing formation conditions, the performance (viscosifying ability, thermal stability, and oil displacement ability) of TVPs was considerably better than that of conventional HPAM polymers [128]. A comparison between the viscosities of TVPs and HPAM is shown in Figure 12 [128]. In Figure 12, the shear rate was 7.34 s^−1^. After the critical temperature was reached, TVPs had a considerably higher viscosity than HPAM. The structure of TVPs is available in [128]. Core flooding tests showed that TVPs have higher incremental oil recovery than HPAM under comparable conditions. Regarding the injectivity of TVPs, the tests showed that the RRF and RF of TVPs were higher than those of HPAM, but within a reasonable degree. The RRF and RF values implied that there was a low risk of blocking and that the polymer has a good mobility reduction ability. TVPs are not currently produced on an industrial scale.

## 4. Field Tests and Commercial Practices

**Figure 13:**
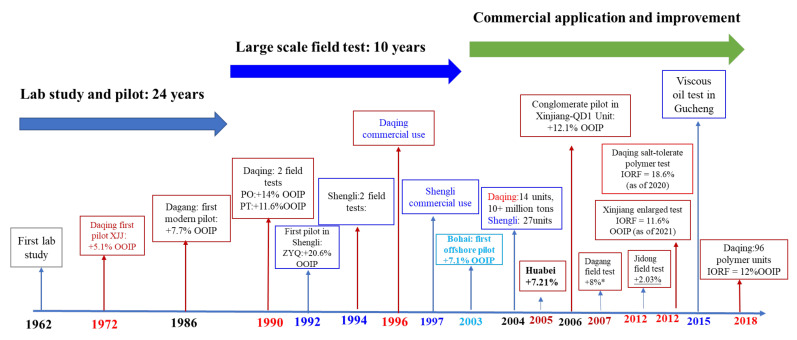
Polymer flooding in China (revised from [59]).

The first small-scale pilots of polymer flooding were conducted in Dagang and Yumen in the 1960s [165]. The first laboratory screening of polymer flooding was carried out in 1965, and the first polymer-flooding pilot was implemented in Daqing in 1972 [56]. However, the polymers used were not modern HPAM. Polymer-flooding field tests were conducted on a larger scale than the pilot in Dagang (1986), Shengli (1992), Bohai (2003), and Xinjiang (2006). The polymer-flooding history of China is summarized in Figure 13, which was revised from [59]. The asterisk in Figure 13 represents the predicted IORF. The industrial field tests performed in China are semicommercial and make use of a large number of wells. The main goal of polymer-flooding research varied according to different reservoir development conditions. For example, despite the fact that polymer flooding was commercially used in Daqing in 1996 [166], several polymer-flooding field tests were conducted to verify its suitability in less permeable formations, such as third-class layers (TCLs) [7], or to verify if the SRPs were prepared with produced water [162,163]. According to recent research, 96 blocks in Daqing have commercially used polymer flooding, and as of the 31st of December 2017, polymer flooding accounted for 23% of the total oil production [167]. The average IORF of all industrial application units was 12% of the OOIP [166]. Recent advances indicated that the IORF of polymer flooding in the second-class layer (SCL) was higher than 12% of the OOIP [164]. This is a promising result because the SCL was more difficult to develop than the first-class layer (FCL), which has a higher permeability and formation thickness. The annual amount of oil recovered using polymer flooding in China is more than 12 million tons (approximately 210,410 bbl per day) [168]. As the largest chemical flooding application unit, the Daqing oilfield has produced more than 19 million tons of oil by polymer flooding and alkali-surfactant-polymer (ASP) flooding in the past 19 years (as of September 2021) [164]. We summarized the field progress of polymer flooding from two aspects, namely the injection technology and field performance.

**Injection technology**. Two ways of polymer injection were used, namely a centralized preparation and dispersion injection (CPDI) method [169] and a combination method of preparation and injection. CPDI is cheaper compared with CPD. Improved CPDI is used in Daqing as a considerable amount of research has been carried out to optimize its effectiveness to reduce costs [167]. Separate-layer polymer-injection technology (SPT) was also very notable in Daqing because it helps to improve the injection profile [44]. A specialized instrument, namely a pressure blanking plug, was employed in SPT [170]. This device can change the polymer viscosity by elongating and shrinking the molecular chain of polymers. Another key innovation is the eccentric separate injection string, which could significantly increase the relatively low-permeability layer injectivity [170]. Lifting technology [171] has also been employed to improve polymer flooding compared with water flooding. Some field tests showed that polymer alternation water injection technology (PAIT) [165] increased the lower-permeability-layer fluid intake compared with the traditional one slug injection scheme. This could especially be important for the HCHMW, which are at a high risk of blockage formation. PAIT may be further improved because the injected water slug could dilute the polymer slug, which could reduce the benefit of mobility control.

## 5. Typical Field Tests and Applications

### 5.1. Shengli Oilfield Polymer Flooding

The polymer-flooding pilot in the Gudao ZENZ block in Shengli was performed in 1992 [172]. An expanded test was conducted in 1994, which led to a development of commercial application of the 70 °C reservoir. Another polymer flooding using KYPAM in the Shengtuo No.1 block was conducted in 2002. A commercial expansion use was implemented in 2005 [172]. The success of the polymer-flooding tests led to the commercialization of polymer flooding in Shengli in 1997 [173]. As of October 2005, 22 units in Shengli used polymers to keep oil production stable [8]. As of 2012, more than 60 blocks have been used for polymer or SP flooding [172]. A survey on polymer flooding in Shengli indicated that different units had different IORFs [173]. It is theorized that a mobility ratio of water–oil in the range of 2–7 would be sufficient to have a prominent influence on the IORF and development effect [173]. Produced water composition can seriously harm polymer viscosity. Due to the presence of Fe^2+^ and H_2_S in waste water, well-head polymer solution viscosity decreased from 20 to 1–5 cP [174]. High-permeability streaks led to the production of polymers that had the same concentration as the ones that were initially injected [172]. The sulfide concentration in produced water from Shengli is 5–6 ppm [174] and is sufficient to reduce the polymer solution viscosity from 200 to 50 cP [172]. The reservoir geology and remaining oil saturation affected the polymer-flooding results [173]. A new SP-flooding field test based on a carbon footprint reduction model was conducted in an offshore reservoir owned by the Shengli oilfield [175], which could be very promising in a time where carbon neutrality is so highly valued.

### 5.2. Xinjiang Oilfield Polymer Flooding

The polymer flooding pilot was conducted in a conglomerate reservoir in the QD1 block of the Xinjiang oilfields [165,176]. This was the first reported instance of conglomerate reservoir polymer flooding being implemented in China. This test has nine injectors and 16 producers, with a five-spot well pattern. The well spacing was 200 m. Among the 25 wells, 19 were newly drilled. The KYPAM (HJKY-2) polymer was used in this test. Responses were detected for all 16 of the producers. As of December 2015, an IORF of 12.1% was reported, which is higher than what was previously predicted. When the new wells started to produce oil, the total water cut was 92% [165]. After the reference water flooding, the water cut was 95.6%, which made it uneconomic for this purpose. After the polymer flooding, the water cut decreased to a minimum of 65%. An enlarged commercial test with 276 wells was implemented in 2013.

Below are the key conclusions and observations:

(1) The selection of polymer viscosity for conglomerate polymer flooding requires further investigation.
(2) The injection of highly viscous polymers is risky. The conclusion that ultrahigh MW polymers could be applied to conglomerate reservoirs should be accepted with caution [176].
(3) The notion that the use polymers for profile control could reduce formation blockage is particularly important for polymer flooding.
(4) Polymer viscosity loss at the well head is 7.8% compared with other dilution sites, which is considerably lower than the results from other Chinese oilfields.
(5) The water-cut performance is considerably different from those of other polymer-flooding field tests with regard to drastic changes and slow water increase [165]. It remains unclear whether this is caused by high heterogeneity or frequently used fracturing measures.

### 5.3. Gucheng Oilfield Polymer Flooding

Ultrahigh-MW (35 MDa) polymer-flooding field tests were conducted in the Gucheng oilfield at the Mi 125 unit [177,178]. The Gucheng oilfield is part of the Henan oilfield. The concentration of the injected polymer is 2000 ppm. Twenty-two wells were injected with polymers at concentrations of 2200 (viscosity of 167.6 cP) and 2500 ppm (213 cP) [179]. This resulted in an increase in the average injection pressure from 5.1 to 8.6 MPa. The well-head polymer concentration was 2285 ppm with a viscosity of 144 cP. A 0.224 PV polymer was injected. Positive responses were detected for 27 producers, which accounted for 65.8% of the total number of producers. The produced polymer concentrations ranged from 821 to 1382 ppm [9]. The average polymer concentration of the produced polymers in the 40 wells was 821 ppm [179]. Polymer channeling was partially caused by the great layer permeability and irregular well spacing [179]. It is, as of yet, still unknown if the test results were as good as predicted.

### 5.4. Bohai Oilfield Polymer Flooding

**Associative polymer flooding**. Associative polymers, such as HAWPs, have been successfully used in an offshore reservoir in the Bohai oilfield since 2003 [118,154]. Commercially used HAWPs that have been field-tested include AP-P4 in SZ 36-1 [118] and AP-P3 in Dagang [180]. An inverse nine-spot pattern with a well spacing of 350–600 m was adopted in the Bohai oilfield. The pilot included only one injector and an enlarged well-group test with 11 injectors and 33 producers [181]. The authors of [180] reported that associative SP flooding was conducted in an onshore reservoir in Dagang, and some of the blockages that occurred were reported by [46,158,182,183,184]. The unblocking of the injectors has been of great concern for operators in recent years. Since June 2019, the IORF of polymer flooding in the Bohai oilfield was reportedly more than 7.1% of the OOIP [118]. It was estimated that the total polymer-flooding-block input–output ratio (IR) was 1:3.7 in 2010 [185], which is considerably lower than that of the commercial polymer-flooding IR of 1:6:83 in Daqing [186]. However, this is not an accurate comparison because of the harsher offshore reservoir conditions. The high-viscosity associative polymer AP-P4 was injected into the offshore Bohai reservoir. An SP-flooding test conducted in the Dagang oilfield has reportedly very high injection pressures [180]. Although early injection of the polymers improves the sweep efficiency at the pore scale [36], it remains unknown whether polymers with the same high viscosity as for high water-cut injections could be injected. No significant decreases in the water-cut “funnel” were observed in an early polymer-injection field test conducted in the offshore LD unit, which is inconsistent with other studies [187]. Methods for evaluating the effectiveness of associative polymer flooding in offshore reservoirs are still under investigation. A blockage phenomenon was reported in some wells, but the blockage mechanism remains unknown. The lower polymer concentration in produced water could account for the occurrence of the blockage.

**IAJN flooding**. The IAJN pilot started in August 2011 and ended in May 2013. The monthly oil production increased from 373.59 to 1200.06 m^3^ in October 2011. The fluid and oil production increased, and the water cut decreased with the injection of IAJN. From September 2012, no polymers were injected for approximately 2 months because of the weather and facilities maintenance issues, which resulted in an increase in the water cut and a decrease in oil production. In addition, well blockage was reported [188].

### 5.5. Daqing Oilfield Polymer Flooding

Polymer flooding was commercially used in Daqing in 1996. As of December 2017, the cumulative oil production is 0.219 billion ton (1.6 billion bbl). The average IORF of all commercial blocks is 12% of the OOIP [166]. A survey on the first 50 FCLs and 22 SCLs indicated that the current IORF is 13.5% of the OOIP, whereas it was predicted to be 14% of the OOIP [165]. The current polymer utility factor is 54 tons per ton [165]. Experiences and practices in polymer flooding in Daqing have been well-reviewed [42,43,44,45]. Polymer flooding has been commercially applied in Daqing for 20 years. Current polymer-flooding research in Daqing is mainly focused on reducing costs and expanding its use to lower-permeability and thin-layer applications, which are the main characteristics of TCLs [189]. A recent field test on TCLs indicated that the IORF of polymer flooding could be 4% of the OOIP [7]. The IORF in the low-permeability layer, namely the TCL, by polymer flooding is higher than 5% of the OOIP [190]. In this section, we summarized four typical field tests, namely polymer alternate injection, HCHMW, APPs, and SRPs with produced water.

#### 5.5.1. Polymer Alternating Injection Technology

PAIT is used to enlarge the relatively low-permeability-layer fluid adsorption amount by creating a pressure change between the high- and low-permeability layers [165]. The MW and concentration of the polymers were changed during polymer injection to avoid polymer flowing in the high-permeability layer. The main concept of this method is centered around the injection of the high-viscosity slug into the high-permeability layer and the injection of the low-viscosity slug into the low-permeability layer. According to a numerical simulation, when the polymer dosage is decreased by 25%, a higher recovery is achieved when the injections are alternated from one to six small slugs than when one big sum slug is injected. Four alternation injection field tests were conducted in 2011, wherein the HCHMW injection was used as the basic scheme. Detailed information of these tests was not available.

#### 5.5.2. High-Concentration High-Molecular-Weight Polymer-Flooding Field Tests in Daqing

A number of HCHMW field tests and commercial applications [107] were conducted in Daqing because of the theory that polymer viscoelasticity could reduce ROS and improve displacement efficiency [60,62,63]. The HCHMW is regarded as a potential solution for postpolymer flood reservoirs [191,192]. The IORF in the HCHMW is reportedly as high as 20% of the OOIP, which is higher than those in ASP-flooding field tests. The MWs of the polymers that the HCHMW consist of are typically 20–25 MDa [192] and sometimes 35 MDa [193]. Industrial polymer flooding in Daqing mainly uses polymers with medium MWs of 12–19 MDa [193], although high-MW polymers are more popular because of their high solution viscosity. Before 2008, the polymer concentrations used in Daqing field tests ranged from 1000 to 1300 ppm. From 2008 to 2016, the polymer concentration was increased to 2000–2500 ppm [26]. The typical polymer concentrations used for HCHMWs are 2500 ppm [192] and sometimes 3500 ppm [191]. The injection rate varied from 0.1 [194] to 0.25 PV per year. The slug size was often 0.5–0.7 PV. The well spacing varied from 106 to 144 m [195]. As HCHMWs have been well-documented, we mainly discussed some of the recent updates in the field and observations regarding the difficulties associated with the injection process.

**Observations**. Many problems are associated with the use of HCHMWs.

(1) *High injection pressure.* The injection pressure of 20% of the injectors is higher than the formation parting pressure [18]. High injection pressure is often a sign of blockage [111]. Although induced fractures could contribute to the improvement of the injectivity [26], uncontrolled fracturing could have unknown consequences.
(2) *Formation blocking.* Polymer blocking is reported in many Chinese oilfields [183,188]. As of 2004, approximately 12% of conventional polymer-injection wells experience poor injectivity or a severe decrease in injectivity in the Daqing oilfield [196]. As polymer flooding was first conduced in the FCL, which has a high permeability and thick layer, the injectivity well ratio is considerably higher for SCLs and TCLs. When polymers with MWs of 25 MDa and concentrations of 1000 ppm are injected into the FCL in block A, 50% of the wells could not be continuously injected, and the injection rate was significantly affected [106]. Even when measures such as fracturing and surfactant injection were taken, the injectivity could not be improved.
(3) *Pump damage.* In a 125 m well-spacing HCHMW unit, the injection pumps experienced frequent issues [197]. From January to September 2013, only 51.8% of the scheduled polymer amount was injected, and the injection volume was limited to 67.8% due to the pump problems. Fifteen injectors could not be injected as scheduled, which accounted for 62.5% of the injectors and affected 38.8% of the producers.
(4) *Production loss.* The fluid production loss is significant in HCHMWs [198].
(5) *Well damage.* Recently, full-block well-casing damage has been reported in some blocks [199]. Well-casing damage caused huge economic loss [200]. The casing damages experienced in Daqing could be attributed to a number of causes, including the high injection pressure [200]. A survey on the Daqing oilfield indicated that the correlation between the well-casing-damage ratio and injection pressure was in good agreement [201].
(6) *Production of fluid treatment*. It takes longer time to separate oil and water for HCHMWs. The reduction of produced fluids could be a potential focus of a future study [166].

**Discussion**. **Is HCHMW widely promoted?** Many years have passed since the viscoelasticity theory was proposed in 2000 [60], and the first HCHMW pilots were conducted in 2002 [193]. As HCHMW field tests have reported IORFs of 20% in FCLs, which are as high as those of ASP flooding [202], it was expected that the use of HCHMWs for FCLs and some SCLs would have replaced ASP flooding. ASP flooding has been commercially used in Daqing since 2014 [166,202]. One key reference in 2019 reported that there are a limited number of applications of HCHMWs in Daqing [56]. This does not agree with the common consensus that high-concentration polymer flooding is widely used in Daqing [26]. HCHMWs are likely used in postpolymer flood technologies [56,191,192]. An unusual result was obtained when 200–300 cP HCHMW polymers were injected, namely the pressure increased by only 2–3 MPa with concentration polymers, which increased by 40–50 cP. Except for channeling, unnoticed fracturing during polymer flooding [26] could be accounted for the small increase in injection pressure. It is still being debated whether the IORF results of HCHMWs were caused by an increase in the sweep or displacement efficiencies resulting from the polymer viscoelasticity. When the contributions of intended and unintended fracturing, as well as infill drilling, are excluded, it is unknown whether the drastic decrease in water cut was caused by an increase in the displacement efficiency. However, the drastic decrease in water cut observed in many field tests at high water-cut stages is likely caused by the fast sweep efficiency increase rather than the slow displacement efficiency. Further investigation will be required to prove or disprove this theory.

#### 5.5.3. Salt-Resistance Polymer-Field Tests in Daqing

**Figure 14:**
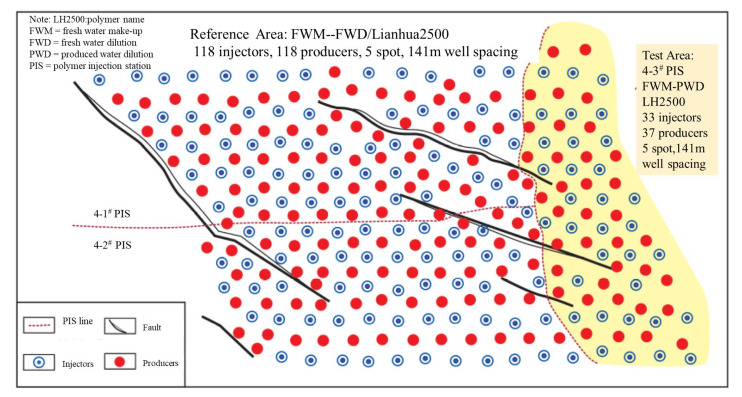
Polymer-flooding well pattern of test and reference areas (revised from [134]).

**Figure 15:**
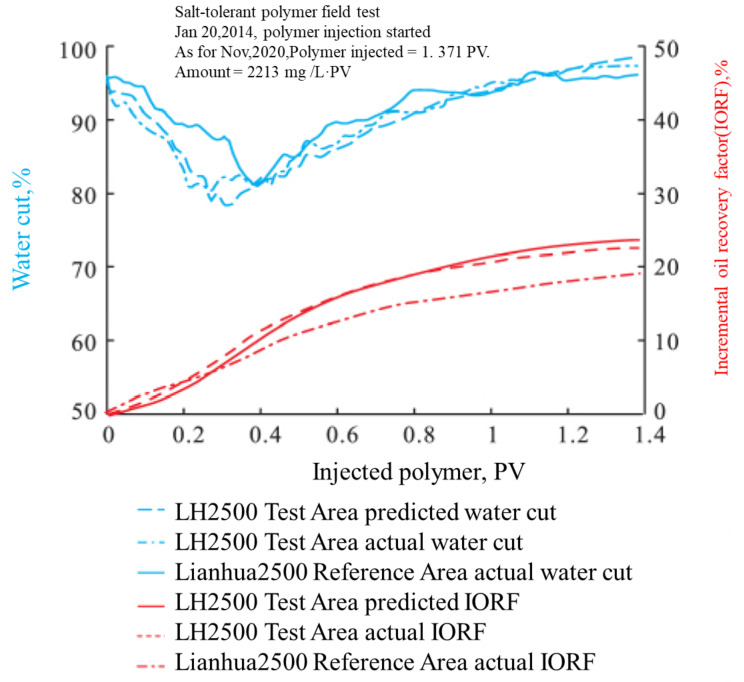
Polymer-flooding performance (revised from [134]).

As for June 2019, 80% of the polymer-flooding units in Daqing used produced water to prepare the polymer solutions [163], and new SRPs were developed and field-tested [135,136,162,163]. As of March 2019, SRP DS800 was field-tested in a weak ASP-flooding field test, which had nine injectors and 16 producers [135]. The average formation permeability was 96 mD. Recent advances indicated that DS800 was industrialized in Daqing, and an increase in the incremental oil recovery was noted as a result of the saved polymer dosage that was up to 20% [164]. For DS2500, 3.5 tons of polymers were produced as of 2019 [135], and it was industrially produced and injected in Daqing [164]. However, no data from field tests were available. SRP LH2500 field tests in the X6Z unit were introduced in a paper by [134]. The field performance was shown in Figure 14 and Figure 15 [134]. At a polymer-injection amount of 1878 mg L^−1^ PV^−1^, the current IORF was 17.4% of the OOIP, and the final predicted IORF was 19.6% of the OOIP, which is 6.6% higher than the comparable block used in conventional polymers (Lianhua2500) with produced water. The recovery was also 1.1% higher than the polymer-flooding block that used fresh water to make and dilute the polymers [162]. This was a large-scale field test. Although some geology uncertainties exist between the two regions in Figure 14, the IORF difference was excellent for this large-scale field test with 70 wells. Another SRP field test was reported [136].

#### 5.5.4. APP Field Tests in Daqing

APPs are another amphiphilic polymers that have drawn a considerable amount of attention in Daqing. A laboratory evaluation of APPs was recently released [127,149]. Many APP field tests have been conducted in Daqing using different formation types [127,151,152]. An IORF of 12% of the OOIP was recently reported for a TCL [203]. The IORF of an FCL in Daqing was as high as 28.7% of the OOIP [127]. The FCL IORF of APP flooding from postpolymer flooding pilots and large-scale field tests verified that the IORF could be 10% of the OOIP or even higher [151,152]. An SCL field test conducted in Daqing reported staged and predicted IORFs of 4.9% and 13.1% of the OOIP, respectively [204]. To solve the problem of productivity loss, fracturing was widely employed in nearly all APP-flooding tests.

### 5.6. Discussion on Practical Issues

**Are higher injected polymer viscosities better than lower ones?** Encouraged by the mobility control theory and polymer viscoelasticity effect on ROS reduction, many researchers from China were in favor of viscous polymer flooding, wherein polymers with very high viscosities are injected. In conventional polymer flooding conducted in Daqing, the injected well-head polymer viscosity is four to five times larger than the viscosity of underground oils. In HCHMW practices, this viscosity ratio can be as high as 30 times larger [26]. For heavy-oil recovery in Shengli, the viscosity ratio is less than unity because such high-viscosity polymers are not economically available under high-temperature conditions. Other than this drawback, a high polymer viscosity is desired. However, studies [25] and practices on heavy-oil polymer flooding in North America indicated that the optimal polymer viscosity is much lower than what many researchers had predicted. The latest HCHMW practices in Daqing [56] and other oilfields [111] in China indicated that the viscosity of the injected polymers should not be as high as possible because too high viscosity is wasteful and could have detrimental effects.

**Are larger injected polymer slugs better than lower ones?** Although surveys on polymer-flooding field tests do not indicate a trend where higher IORFs are associated with larger polymer slugs, there are many occasions where higher IORFs are attained at lower polymer slugs (Figure 3) [1]. The key is oil saturation before polymer flooding and heterogeneity caused by high water cuts. Seven field tests in Daqing showed that approximately 88.37% of oil is produced from polymer-injection stages [205], whereas postwater-flooding oil production accounted for 60% of oil production in the Shengli oilfield [206]. The slug size grew from 240–380 to 640–700 mg L^−1^ PV^−1^ during industrial polymer flooding in Daqing [43]. The optimal polymer slug size is approximately 0.7 PV [43]. Although the effects of using polymer slugs with large sizes and at high dosages were investigated, the latest results indicated that these tests did not have the desired effects [56]. The optimal economic polymer dosage in one block in Shengli was 500 mg L^−1^ PV^−1^ [206]. Hence, the use of large injection polymer slugs is not advised.

**Should the polymer-injection pressure be lower than the formation parting pressure?** Injections at pressures greater than the formation parting pressure and fracture extension do necessarily produce detrimental effects [26]. The induced fracture orientation and length are important issues that require further investigation. Another important issue is the well-completion quality. The maximum injection pressure is influenced by the injection rate, well spacing, porosity, and apparent-water-intake index [43]. In polymer-flooding practices, many fractures are induced without being noticed. If the wells are old and the casing quality is not good, potential casing damage associated with high injection pressures could be an issue. The authors of [207] showed the casing damage well number along with the average injection pressure. A correlation between the casing damage well number and injection pressure could be noted. When measures were taken to reduce the water injection pressure, the casing damage well number was significantly reduced.

**Is polymer adsorption important for EOR?** The classic screening criteria require polymers used in EOR to have a low adsorption into formation rocks. However, current amphiphilic polymer-flooding field tests in Daqing and Bohai are inspiring. As amphiphilic polymers have much higher adsorption amounts than conventional polymers, the evaluation of polymer adsorption is brought into question. Many studies in China neglected to mention the influence of polymer adsorption. The formation retention in China was neglected partly because the revised national standard removed the requirements of polymer retention.

**Is it necessary to revise the polymer-flooding capillary desaturation curve?** If viscoelasticity plays an important role in reducing ROS, as is shown in Figure 6 [32] and Figure 7 [39], it is necessary to revise CDCs. Figure 6 and Figure 7 require the injection of HCHMW polymers for a higher viscoelastic effect on ROS. However, further research is required to adequately explain the influence of the viscoelasticity effect on ROS. The viscoelasticity effect was not present in the polymer flooding of heavy oils [25]. The latest research on polymer flooding in Daqing [26] provided less support for the viscoelasticity effect on ROS reduction. Furthermore, the gravity effect may contribute to the low ROS in Figure 7 [39]. Although the classic capillary number was widely accepted, it is necessary to restudy this theory [49].

## 6. Postpolymer-Flooding Enhanced Oil Recovery Techniques

**Figure 16:**
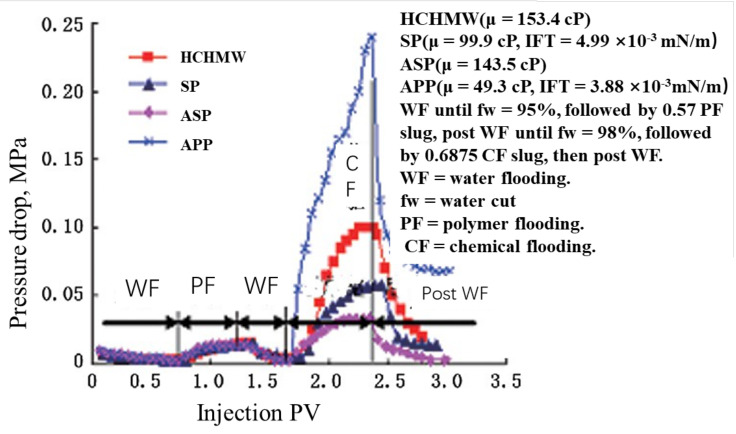
Injection pressure of postpolymer-flooding enhanced oil recovery techniques (revised from [132]).

Approximately 50% of the OOIP of FCLs remained according to FCL coring tests in Daqing [151]. The enhancement of oil recovery beyond polymer flooding is of great concern. The Daqing FCL coring tests also indicated that the average permeability increased by 14.78% [152]. Postpolymer-flooding reservoirs become increasingly heterogeneous. Some of the proposed techniques for postpolymer flooding include the heterogeneous phase combination [208,209], SPs, HCHMWs [56], ASPs [116,202], in situ polymer reuse [210], microbial EOR [152,211], APPs [204], and foaming flooding [152]. Special attention should be given to laboratory tests because the results from these tests are sometimes difficult to interpret and can be misleading. For example, in a laboratory study of different postpolymer flooding EOR techniques, the IORF of SP flooding was higher than that of ASP flooding, one slug polymer flooding, and PAIT [126]. However, the field test in Daqing indicated that the performance of SP flooding in postpolymer flooding reservoirs is very poor and that it had a considerably lower IORF than ASP flooding [152]. The IORF of the SP-flooding field test in postpolymer flooding was only 2% of the OOIP [212], which was considerably lower than previously predicted. The author of [152] also reported the importance of infill drilling in postpolymer-flooding EOR techniques. Several EOR techniques were evaluated through core flooding tests, and the injection pressures that were used are shown in Figure 16 [132]. The increase in injection pressure was generally viewed as an indication of an enlarged sweep volume in mature water-flooding field practices. However, the extreme high injection pressures are actually a sign of formation blocking. APPs had the highest injection pressure in Figure 16. Injectivity issues could have arisen if induced formation fractures were not taken into consideration. However, the actual field injection of such polymers could cause induced fractures, which could have a positive effect if the fracture orientation is favorable [26]. In other words, the high injection pressure in laboratory tests does not necessarily represent poor injectivity in field tests. Field pilots were necessary, and their results needed to be properly explained. There exists a risk of well damage due to high water injection pressures, as verified by field practices in Daqing. A promising EOR technique for postpolymer flooding reservoirs is HCPF, which can be regarded as a combination of preformed particle gel and SP flooding [213]. HCPF has been field-tested and commercially used in the Shengli oilfield [209]. HCPF field tests have also been performed in Daqing. More information regarding the polymer-flooding field tests and practices in water-flood and polymer-flood reservoirs have been provided elsewhere (Guo et al.,2021).

## 7. Polymer Flooding in Future

**Figure 17:**
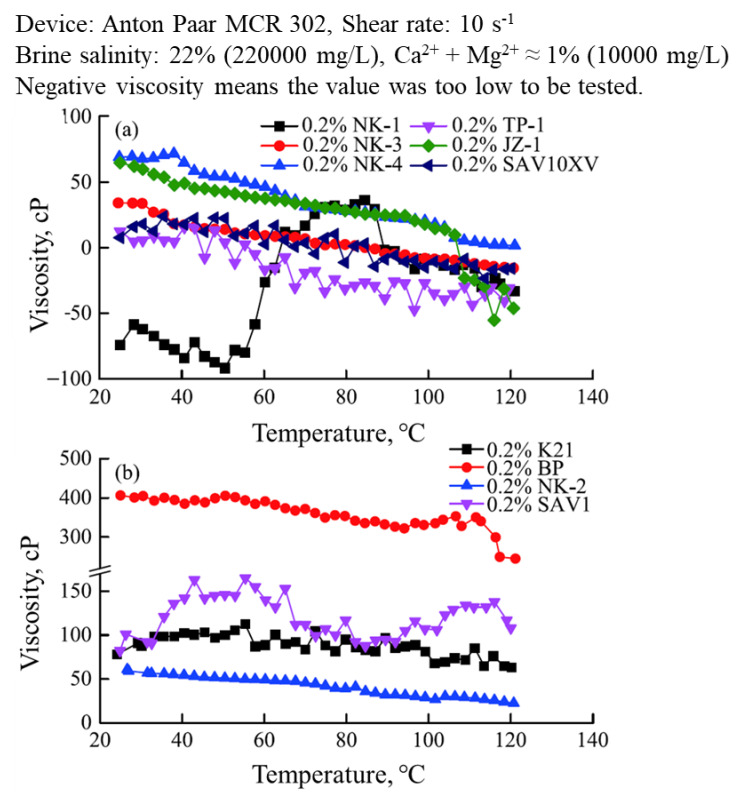
Polymers screened for high temperature and high salinity in Tahe oilfield (revised from [129]).

Polymer flooding at high-temperature (80–160 °C) and high-salinity (300,000 TDS or higher) reservoirs is still under development [128,129]. Figure 17 shows 10 different types of chemicals screened for use in the HTHS Tahe reservoir, which has a formation temperature of 100–160 °C, formation salinity of 220,000 mg L^−1^, and divalent cations at concentrations of 10,000 mg L^−1^. The structures of the chemicals and polymers are not provided in this paper. The negative viscosity in Figure 17 was caused by the values being too low, which made the instrument report negative values. Figure 17 shows a potential solution for chemical flooding in HTHS reservoirs. Although the viscosity of the polymer solutions was the significant parameter, many other features, including thermal stability, injectivity, transportation, and adsorption, should also be taken into account. The polymer flooding that occurred in the low-permeability sandstone and carbonate reservoirs [16,214,215,216] was not technically mature yet, although great advances have been made in this regard [130]. Research on polymers that displace very viscous oils should be creatively conducted. For example, the combination of polymer flooding with horizontal wells could produce unexpectedly good performance, which has been well-practiced in the Pelican Lake [13,14,15,16,17] and Alaska North Slope [22,23,29,217]. Hybrid methods [20,82], such as the combination of polymer flooding with LSW, could also be viewed as a promising alternative. The potential of polymer flooding in conventional reservoirs can be significantly improved as polymer-flooding oil production only represents 12% of the total oil production for China National Petroleum Company (CNPC) [218] and 34% in the Daqing oilfield as of 2019 [164]. As polymer flooding is a potentially promising way of reducing the carbon footprint [40,41], it is expected that the use of polymer flooding will increase worldwide.

## 8. Conclusions

1. Polymer flooding has been field-proven to be a good EOR technique in China. Domestic polymer production technologies contributed to the cost reduction of polymer flooding in very-large-scale commercial applications. New polymers are under development that could improve oil recovery performance and reduce cost.
2. In addition to increasing the sweep efficiency by injection of viscous polymers, many Chinese researchers believe that the viscoelasticity of HPAM also positively influences displacement efficiency. The viscoelasticity effect of polymers on displacement efficiency theoretically supports injecting the most practical high-viscosity polymers available in China with a much higher viscosity than required in conventional mobility control.
3. There are still different opinions on the viscoelasticity effect of polymers on reducing ROS. Insignificant ROS reduction reported in many core flooding tests may be caused by heterogeneity due to a microscopic sweep efficiency increase, experimental artifacts, or mechanisms such as wettability change effects and low-salinity effect.
4. Comb-shaped KYPAM has desirable viscosifying and other EOR properties such as adsorption and cost. Amphiphilic polymers have attracted increased attention in recent years in China. HAWPs have had few field tests in onshore reservoirs but many applications in offshore reservoirs in China. It shows good salt-resistance performance under seawater-injection brine conditions. The retention of polymers into formation was not given enough attention in China, which may lead to formation plugging.
5. There does not exist a consensus on the ideal method of injecting highly viscous polymers into the formation without blocking in China. Different matching relationships between polymers and formation permeability were determined using different theories. Although high polymer-injection pressure may lead to increased sweep efficiency, it may also be a sign of blockage and can cause very serious well damage.
6. Polymer flooding in the offshore Bohai reservoir has been reported to have plugging problems. How to solve or avoid the plugging, when to inject polymers, and what viscosity to select for offshore reservoirs remain to be further investigated.
7. Although HCHMW polymer field tests have reported a much higher IORF than conventional polymer flooding, it has not been extensively applied in China up to present. This fact deserves special attention.
8. Whether high injection pressure of APPs is a sign of poor injectivity in field practices remains to be evaluated. This may be due to the induced fractures, which were not seen in laboratory studies.
9. Polymer-injection technology progress, such as separate layer injection and low-viscosity loss process, helps to increase oil recovery and reduce polymer-flooding cost, which can be lower than water flooding according to practices in China.
10. Many EOR techniques have been tested in reservoirs after polymer flooding. HCPF, amphiphilic polymer, HCHMWs, and ASP flooding are potential EOR techniques. HCPF was the field-proven choice for reservoirs with high heterogeneity and is a mature EOR technique in postpolymer-flooding reservoirs in Shengli.
11. As polymer flooding was found to reduce carbon footprint by researchers, wider application, both in conventional and harsh reservoirs such as low-permeability and HTHS reservoirs, can be expected.

## Figures and Tables

**Table 1:** Polymer solution viscosity loss [88].

Sampling Place	Preparation Site	Injection Well Head	Wellbore Perforation Hole	105 m Sampling Well
Viscosity (mP s)	12.3	7.7	4.4	4.1
Viscosity loss (%)	0	37.3	64.2	66.7

## Data Availability

Not applicable.

## Nomenclature

APP	amphiphilic polymer 1
ASP	alkali-surfactant-polymer
CAC	critical aggregation concentration
CDC	capillary desaturation curves
CDG	colloidal dispersion gel
CPDI	centralized preparation and dispersion injection
CNPC	China National Oil Company
DLS	dynamic light scattering
EOR	enhanced oil recovery
FCL	first-class layer in Daqing oilfield
HAWP	hydrophobically associating water-soluble polymer
HCHMW	high-concentration high-molecular-weight polymer flooding
HPAM	hydrolyzed polyacrylamide
IAJN	amphiphilic polymer 2
IORF	incremental oil recovery factor
IR	input–output ratio
KYPAM	comb-type polymer
LSP	low-salinity polymer flooding
LSW	low-salinity water flooding
MW	molecular weight
OOIP	original oil in place
PAIT	polymer alternation injection technology
RF	resistance factor
ROS	residual oil saturation
RRF	residual resistance factor
SCL	second-class layer in Daqing oilfield
SP	surfactant-polymer
SPT	separate-layer polymer-injection technology
SRP	salt-resistance polymer
TCL	third-class layer in Daqing oilfield
TVP	thermoviscosifying polymer
